# Repurposing a pore: highly conserved perforin-like proteins with alternative mechanisms

**DOI:** 10.1098/rstb.2016.0212

**Published:** 2017-06-19

**Authors:** Tao Ni, Robert J. C. Gilbert

**Affiliations:** Division of Structural Biology, Wellcome Trust Centre for Human Genetics, University of Oxford, Roosevelt Drive, Oxford OX3 7BN, UK

**Keywords:** astrotactin, BRINP, evolution, perforin-like protein

## Abstract

Pore-forming proteins play critical roles in pathogenic attack and immunological defence. The membrane attack complex/perforin (MACPF) group of homologues represents, with cholesterol-dependent cytolysins, the largest family of such proteins. In this review, we begin by describing briefly the structure of MACPF proteins, outlining their common mechanism of pore formation. We subsequently discuss some examples of MACPF proteins likely implicated in pore formation or other membrane-remodelling processes. Finally, we focus on astrotactin and bone morphogenetic protein and retinoic acid-induced neural-specific proteins, highly conserved MACPF family members involved in developmental processes, which have not been well studied to date or observed to form a pore—and which data suggest may act by alternative mechanisms.

This article is part of the themed issue ‘Membrane pores: from structure and assembly, to medicine and technology’.

## 1. Introduction

Pore-forming proteins are well known for their roles in pathogenic attack and immunological and other forms of defence, as several of the reviews in this Special Issue attest. As noted elsewhere, the membrane attack complex/perforin and cholesterol-dependent cytolysin family (the MACPF/CDCs) belong to a pore-forming superfamily of proteins, which form giant pores on membranes by penetration and with the capacity to induce severe damage. The mechanism of pore formation by MACPF/CDC proteins has been thoroughly studied and well established [1–4]. However, what has been less intensively studied but is becoming increasingly understood is that MACPF/CDC proteins are also important players in development [5,6], although whether these developmental homologues of perforin, complement proteins and bacterial toxins are actually themselves pore-forming proteins is unknown.

In this review, we will discuss primarily the developmental and likely non-pore-forming roles that MACPF/CDC proteins play. We will first give a brief overview on the structure and mechanism of pore formation by MACPF/CDC proteins and then describe their distribution. Then we will focus on the structure and function of MACPF proteins in vertebrates, and especially in humans, that are involved in development.

## 2. Structure of membrane attack complex/perforin proteins and mechanism of pore formation

**Figure 1.**
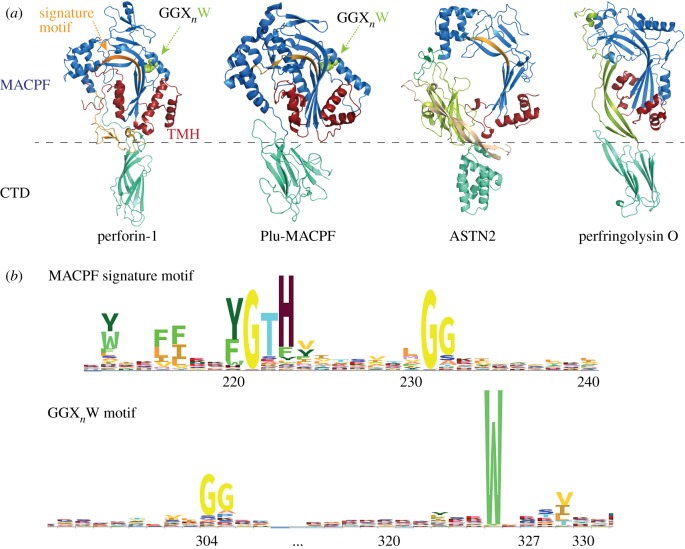
Structure and sequence conservation of MACPF proteins. (*a*) Structure of representative MACPF/CDC superfamily proteins: murine perforin-1 (PDB code: 3NSJ), Plu-MACPF of *P. luminescens* (PDB code: 2QP2), human ASTN2 (PDB code: 5J67) and bacterial perfringolysin O (PFO, PDB code: 1PFO). The MACPF domains are coloured in blue with the transmembrane helix hairpins (TMHs) highlighted in red. The MACPF signature motif (Y(F)-G-T(S)-H-X_7_-G-G, where X is any amino acid) which maps to one of the central β-strands is coloured in orange. The other conserved region (GGX_*n*_W) maps to a helix (pointed with a green arrow), and the conserved tryptophan residue with its side chain facing towards the centre of the molecule is shown as green spheres. The C-terminal domains (CTDs) are coloured in aquamarine. ASTN2 lacks the conserved GGX_*n*_W region and PFO (representative of cholesterol-dependent cytolysins) has no directly detectable sequence homology to the perforin-like MACPF proteins. The dashed line indicates the topological domain boundaries between the MACPF domain and CTD, to highlight the fact that MACPF proteins generally contain a large head domain (MACPF domain and other affiliated domains) and a shorter CTD region. (*b*) HMM logo representation of the two most conserved regions in the MACPF domain proteins. The MACPF/CDC signature motif features prominent glycine residues. The GGX_*n*_W motif highlights a conserved tryptophan residue. The HMM logo was downloaded from the Pfam website (http://pfam.xfam.org) and the numbering below the motifs is according to human perforin-1 (Uniprot P14222).

MACPF and CDC family proteins had been identified and studied separately until structures of representatives from both subfamilies were resolved. The structures of complement C8α and a bacterial MACPF protein (Plu-MACPF) from *Photorhabdus luminescens* revealed that MACPFs and CDCs belong to one homologous family of proteins [7–9]. MACPF/CDC domains are generally flat in shape, featuring four central twisted β-strands decorated by two sets of helices (termed transmembrane hairpins, TMHs) positioned on either side of the β-sheet (figure 1*a*). Although the overall fold of the MACPF domain is conserved, there are substantial differences between specific cases such as in the degree of bending of the central β-sheet, the lengths and positions of the TMHs, etc. The sequences of MACPF domain proteins vary greatly among different species, with the only identified signature motif being a Y(F)-G-T(S)-H-X_7_-G-G motif (where X is any amino acid), which maps to one of the central β-strands [1,5] (figure 1*a,b*). Interestingly, a highly conserved tryptophan following a tandem glycine motif (GGX_*n*_W) is identified among the MACPF domains involved in pore formation, but is absent in development-related MACPF domains (such as astrotactins (ASTNs), Torso-like (Tsl) and bone morphogenetic protein and retinoic acid-induced neural-specific proteins (BRINPs)) (figure 1*a,b*). This tryptophan is localized to an α-helix, which was observed to shift upwards upon pore formation by the complement membrane attack complex (MAC) [10,11].

For most of the MACPF/CDC proteins studied so far, the only basis for activity properly established is the oligomerization of many subunits in order to generate a pore-forming assembly [11–16]. Binding to a target membrane serves the basic function of concentrating monomeric subunits on a planar substrate, and this is often accomplished by a C-terminal domain of various (so far, β-sheeted) forms following the MACPF domain [4]. Then, a concentration-dependent oligomerization occurs to form a prepore complex before pore formation ensues [1,17]. A large conformational change occurs during the prepore-to-pore transition: the two sets of TMHs either side of the central MACPF domain β-sheet unfurl, insert into the membrane and form a partial or full transmembrane β-sheet barrel.

In general, the mechanism of pore formation for MACPF domain proteins is similar to that of the CDCs notwithstanding minor variations on the common theme. For example, while CDCs are known to screw into the membrane resulting in a contraction of their oligomer from 11.5 to 7.5 nm in height [13], perforin appears to remain standing at full stretch above the membrane surface after the pore-forming TMHs extend [1]. Different MACPF family members display different distributions of oligomeric sizes. Pores formed by perforin-1 and the MAC have a similar size to each other (10–15 nm in diameter and 18–20 subunits) due to the structural similarity of their MACPF domains [7,14]. The pores of CDCs are generally composed of approximately 40 subunits (approx. 40 nm in diameter); and the pore formed by the fungal two-component MACPF pore-forming protein pleurotolysin generally contains 12 or 13 subunits (approx. 8 nm in diameter) [18–20]—smaller even than perforin-1 and MAC pores. Very interestingly, both MACPF and CDC proteins also form functional arciform pores with a similar curvature to pores of a complete ring form, but the relative arc sizes vary [15,21–24].

## 3. Membrane attack complex/perforin proteins involved in pore formation

The MACPF-containing proteins are widespread in Nature and can be found in all domains of life, with 1271 MACPF-containing proteins in the SMART database (http://smart.embl-heidelberg.de), and they are involved in various aspects of biological processes. The distribution and conservation of MACPF and CDC proteins has been reviewed thoroughly by Anderluh *et al.* [5] and others [2–4,6,25]. Discussion has tended to focus on MACPF/CDC proteins involved in bacterial pathogenesis and in immune system mechanisms of defence. Here, we will discuss some examples of MACPF/CDCs involved in defence and attack but which have received relatively little attention.

The apicomplexan parasites *Plasmodium* spp. and *Toxoplasma gondii*, the causative agents of malaria and toxoplasmosis, respectively, encode several perforin-like proteins (PLPs) [26]. Genetic disruption of any of them significantly arrests the life cycle of their producing organisms, leaving the parasites trapped within their specific stage of infection, mainly due to a failure to breach membrane barriers [27–32]. They are therefore analogous for apicomplexans to the role that listeriolysin plays in infection with *Listeria monocytogenes* [33], although the motility of apicomplexans and their multiple cell forms make the functional roles of their PLPs more complex.

In the plant *Arabidopsis thaliana*, four MACPF proteins have been identified from the genome and two of them were shown to be functional in the plant immune system. CAD1 (constitutively activated cell death 1) is preferentially expressed in newly developing organs, like newly grown leaves, and *cad1* mutants show a cell death phenotype which is linked to plant immunity [34]. Loss of NSL1 (necrotic spotted lesions 1) also associates with cell death and a defence response [35]. Functional study of the other two MACPF proteins in *A. thaliana* has not been reported yet. Secondary structure prediction reveals that the MACPF domain of these *Arabidopsis* proteins resembles that of complement C9, and that the conserved C-terminal domain is predicted to be rich in β-strands, like the C-terminal domains of the CDCs, perforin-1 and apicomplexan PLPs (not shown). The exact functions of the plant MACPF proteins are yet to be identified, and it would be insightful to determine whether they form functional pores on membranes.

**Figure 2.**
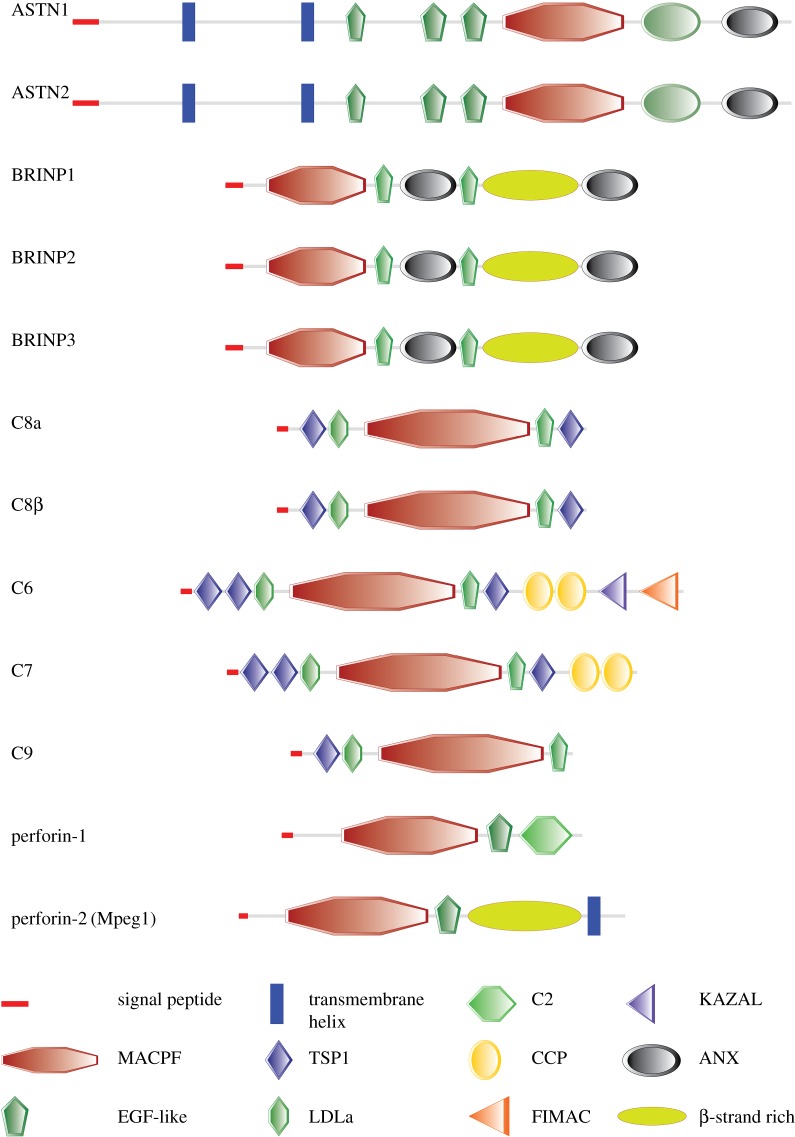
Schematic representation of human MACPF proteins highlighting their domain assembly. Neural-specific MACPF proteins have abbreviated MACPF domains and their MACPF domains are smaller than those of perforins and MAC. The annexin-like domains of the BRINPs are predicted based on their secondary structures and comparison to the structure of ASTN2. The β-strand-rich domains of BRINPs and perforin-2 (ellipses) do not fall into any known domain families. ASTNs and perforin-2 are integral membrane proteins, and their transmembrane helices are labelled with dark blue rectangles. Other domains are depicted following the default settings of the SMART protein database, from where the cartoon representations were downloaded and modified (http://smart.embl-heidelberg.de). FN3, Fibronectin type III; ANX, annexin-like domain; TSP1: TSP type-1 domain; LDLa, LDL receptor class A domain; CCP, Sushi (CCP/CCR) domain; KAZAL, Kazal-like domain; FIMAC, Factor I Module MAC domain.

In the vertebrate immune system, besides perforin-1 and MAC, another highly conserved MACPF protein was recently identified and shown to be capable of forming pores. Perforin-2, initially named as Mpeg1 (macrophage expressed protein 1), was first identified as a gene with expression tightly restricted to macrophages and has subsequently been used as a biomarker for this specific cell type [36,37]. Recently, it was shown that perforin-2 is essential for intracellular defence of parenchymal cells [38,39], and that it restricts the proliferation of the intracytosolic pathogen *L. monocytogenes* [40]. Sequence analysis reveals that perforin-2 is phylogenetically close to the common origin of all MACPF domain proteins, as evidenced also by the wide distribution of perforin-2 (Mpeg1) in sponges, invertebrates and vertebrates [38,41]. Unlike complement components or perforin-1 which are secreted and soluble proteins, perforin-2 is a type I transmembrane protein, projecting its main portion (MACPF domain followed, again, by a β-strand-rich domain) into the endoluminal compartment of endosomes on which it is expressed and with just a short cytoplasmic tail (figure 2). Mechanistically, perforin-2 has been shown to form a pore with a diameter of about 10 nm [39], but the process whereby the lytic activity of perforin-2 is triggered remains to be determined. Perforin-2 was processed into at least two fragments in isolated bacteria after infection [42], and it will be insightful to identify the cleavage site and determine the functional relevance of this cleavage for the control of perforin-2 activity.

## 4. Development-related membrane attack complex/perforin proteins

### (a) Invertebrates: Torso-like

One important MACPF protein involved in development is Tsl, which was initially identified as associated with the localized activation of TOR (target-of-rapamycin) in *Drosophila melanogaster* [43]. Tsl is translocated from the eggshell to the egg plasma membrane, where it enables the regional activation of the Torso tyrosine kinase receptor (Tor) [44]. Ectopic expression of Tsl activates the Tor receptor, leading to developmental abnormality. However, the ligand of Tor receptor is not Tsl itself, but Trunk [45], and Tsl facilitates the local secretion of Trunk [46]. The presence of a MACPF domain in Tsl has led to speculation that Tsl might form a pore to translocate Trunk or promote exocytosis [46].

Compared with other MACPF proteins, Tsl is only composed of a single MACPF domain without an additional C-terminal domain, which makes it exceptional among the PLPs. Sequence alignment and tertiary structure prediction reveal that the MACPF domain of Tsl closely resembles the human immunological MACPF proteins: its two canonical TMHs are a similar size to those in the pore-forming MACPF proteins (data not shown). It remains to be explored whether Tsl can bind to membranes and form a pore. However, biophysical characterization of Tsl has not been achieved due to technical difficulties in obtaining the recombinant protein.

### (b) Vertebrates: astrotactins and bone morphogenetic protein and retinoic acid-induced neural-specific proteins

Four groups of MACPF proteins have been identified in humans, as representative vertebrates: the MAC component proteins (C6, C7, C8α, C8β and C9), perforins (perforin-1, perforin-2), ASTNs and the BRINPs. We now focus on the ASTNs and BRINPs, including description of key features revealed by the first to have its structure solved: ASTN2, a highly conserved protein involved in neuronal development.

**Figure 3.**
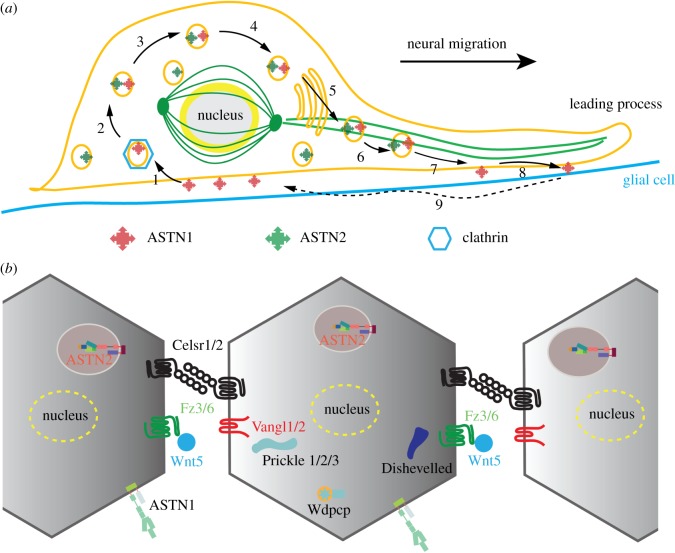
Astrotactins are involved in neuron migration and planar cell polarity (PCP) pathways. (*a*) Schematic of roles played by ASTNs 1 and 2. ASTN1-mediated adhesions undergo endocytosis into clathrin-coated vesicles dependent on ASTN2 (1); the vesicles carrying both ASTNs cycle through the early and recycling endosomes (2–4) and undergo microtubular migration (5–7) until the ASTN1 is re-deposited towards the leading process (7) to form a new adhesion (8) which will be recycled again (9) in step with the cell migration. ASTN1 and ASTN2 are depicted with red and green star-like symbols, respectively, either on the cell membrane or endocytic vesicles. The glial cell membrane is shown as a blue line underneath the neuron cell and the microtubules in green. This image is an updated version of that previously used in [49]. (*b*) Schematic diagram of the PCP pathway. Major components of the PCP pathway include Frizzled3/6, Celsr1/2, Dishevelled, Vangl, extracellular ligand Wnt5, etc. PCP pathway proteins have a typical asymmetrical distribution both inside the cells and on the cell membrane. Vangl1/2 and Frizzled3/6 show polarized distribution on one side of the cells; while Celsr1/2 forming junctions between cells are localized on both sides of cell. The mechanistic roles that ASTNs play in the PCP pathway are unknown. Three polarized cells are shown and for simplicity, the cytosolic PCP proteins (Prickle1/2/3, Wdpcp and Dishevelled) are only shown in the middle cell. The distribution of ASTNs in the polarized cells and how they relate to PCP pathway proteins are not yet known; therefore, their locations are drawn arbitrarily in the cartoon.

Two ASTNs are found in humans, ASTN1 and ASTN2, and both are mainly expressed in neuronal cells in the cerebellum and the cerebral cortex [47–49]. Mice with deleted ASTN1 have a smaller cerebellum, and poorer balance and coordination [50]. ASTN2 has also been identified as a genetic modifier that regulates the global orientation of mammalian hair follicles, revealing a role also in controlling planar cell polarity with the implication of a wider activity in non-canonical Wnt signalling [51] (figure 3). Genetic studies have identified ASTN2 as a risk factor in autism, attention deficit hyperactivity disorder and other neurodevelopmental disorders as well as an earlier onset to Alzheimer's disease [52–54]. The link to Alzheimer's is intriguing and might relate to the implication by others of retromer-association vesicle trafficking in its aetiology [55]. ASTN1 is localized to both the cell membrane and endosomal compartments, and is directly responsible for establishing contacts between neuronal cells and glial cells. Antibody binding to surface-exposed ASTN1 retards the migration of isolated neuronal cells along glial fibres *in vitro* [56]. ASTN2, however, mainly resides in endosomes and expression of ASTN2 leads to internalization of ASTN1 [48]. ASTNs 1 and 2 interact with each other, although the details of the interaction have not been thoroughly characterized [48].

Both ASTNs are integral transmembrane proteins with a molecular weight of about 150 kDa and share more than 50% sequence identity and the same domain architecture: ASTNs contain two transmembrane helices, leaving a large portion of each protein (a small N-terminal domain and large C-terminal domain) in the extracellular region or endosomal compartment, and a separate cytosolic domain which lies between the N- and C-terminal domains (figure 3). The crystal structure of a large portion of ASTN2 reveals that the C-terminal domain contains three EGF-like domains, an MACPF domain, fibronectin type III (Fn(III)) domain and annexin-like domain [57]. The MACPF is abbreviated compared with the canonical pore-forming MACPF domains such as C9 and perforin-1. Pore-forming MACPF domains contain two sets of TMHs with an equal number of residues each, but in the case of the ASTNs, one of the TMHs is 30 residues shorter than the other, so that it would not match it in the formation of a β-barrel (figure 1*a*). The annexin-like domain of ASTNs, although showing no sequence similarity to human annexins, is highly homologous to the annexin repeat fold. The MACPF–Fn(III)–annexin cassette appears folded into a stable unit, with an extensive intermolecular interface between the MACPF domain and Fn(III) domain, further stabilized by C-terminal antiparallel β-strands linking the annexin-like domain with the Fn(III) domain (figure 1*a*). Interestingly, ASTN2 interacts with inositol diphosphate and inositol triphosphate, while ASTN1 does not [57]. The N-terminal domain and cytosolic domain do not match by sequence to any domains of known structure; but considering the high sequence conservation in them, it is likely that they play an important role in ASTN activity.

### (c) Bone morphogenetic protein and retinoic acid-induced neural-specific proteins

BRINPs are another subset of MACPF proteins specifically expressed in the brains of vertebrates. Similar to ASTNs, BRINPs are also predominantly expressed in brain tissues, such as the cerebellum and cerebral cortex [58,59]. BRINP1 was initially identified as a tumour suppressor protein in human bladder cancer, as part of an effort to locate tumour suppressor genes in bladder cancer cells, and was given the name DBCCR1 (deleted in bladder cancer chromosome region 1) [60,61]. Subsequently, it was observed that ectopically expressing BRINP1 caused cell cycle arrest in cultured cell lines such as bladder cancer cells and the NIH 3T3 cell line [62,63], or even mouse embryonic stem cell-derived neural stem cells [58]. Independently, Kawano *et al.* identified that BRINP1 was predominantly expressed in the central nervous system from early developmental stage to adulthood, and that the expression levels of these proteins were greatly enhanced upon stimulation with bone morphogenetic protein or retinoic acid, from which study the name of the proteins was proposed. They also observed that the two orthologues of BRINP1 (BRINP2 and BRINP3) were expressed predominantly in the nervous system, although the expression pattern of individual BRINP genes differed from each other [62]. The absence of BRINP1 in mice causes an increase in hippocampal neurogenesis and behavioural alterations possibly relevant to human psychiatric disorders [64,65].

BRINP3, however, has mainly been studied in non-neuronal tumour cells, and is also known as family with sequence similarity 5 member C (FAM5C). Genetic studies linked BRINP3 to myocardial infarction and aggressive periodontitis [66,67]. It was also shown that BRINP3 was expressed in tongue squamous cell carcinoma and gonadotrope cell pituitary adenomas [68], implying its potential role in tumorigenesis. Intriguingly, BRINPs have been shown to localize to different cellular organisms: in pituitary adenoma cells, BRINP3 was observed to associate with mitochondria [68]; however, in the colorectal adenocarcinoma cell line NCI-H716, it appeared to largely colocalize with the vinculin-rich cytoskeleton. A recent study of the expression pattern of zebrafish BRINPs during development also indicated that they are broadly expressed in the developing nervous system at early stages and then relocated to specific structures, indicating their conserved function in neuronal development [59].

**Figure 4.**
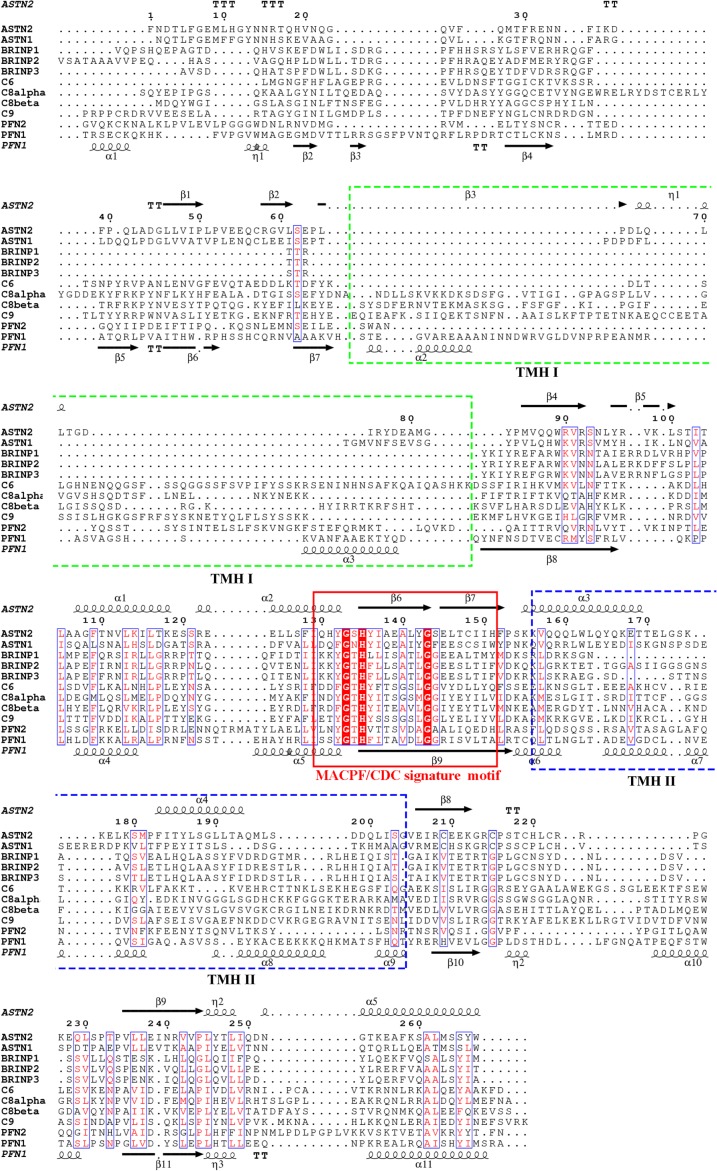
Sequence alignment of human MACPF domains. The MACPF/CDC signature motif is highlighted in red bars with conserved residues indicated. The major sequence difference lies in TMH I: in neuronal MACPF proteins, this TMH is almost missing; while perforins and MAC proteins have an intact helical cluster, matching the size of TMH II. It should be noted that the highly conserved tryptophan in α10 of perforin-1 is absent in neural MACPF proteins (ASTNs and BRINPs).

BRINPs are highly conserved in vertebrates: zebrafish and humans share greater than 90% similarity for BRINP1, greater than 80% similarity for BRINP2 and approximately 80% for BRINP3. All of the BRINPs are predicted to contain a signal peptide, which suggests maturation via the endoplasmic reticulum. Indeed, full-length BRINP3 was glycosylated when expressed in 293T cells. BRINP proteins are smaller in molecular weight (90 kDa) compared with ASTNs (150 kDa). Bioinformatics studies showed that they contain a MACPF domain followed by a potential EGF-like domain (a cysteine-rich domain), and the structures of the domains in their C-terminal regions are unknown. However, secondary structure analysis shows that the C-terminal region contains two short sections rich in helical bundles, matching the secondary structure of the annexin-like domain in ASTNs, which suggests that BRINPs might contain an annexin-like domain as well (figure 2). The MACPF domains of BRINPs are predicted to be similar to those of the ASTNs in terms of the domain size and structure, with one TMH abbreviated and one similar in length to those found in the pore-forming MACPF domain proteins (figure 4). The BRINPs are found on the same chromosome as ASTNs, indicating their mutual origin via gene duplication events over the course of evolution [59].

### (d) Conservation of developmental membrane attack complex/perforin proteins

**Figure 5.**
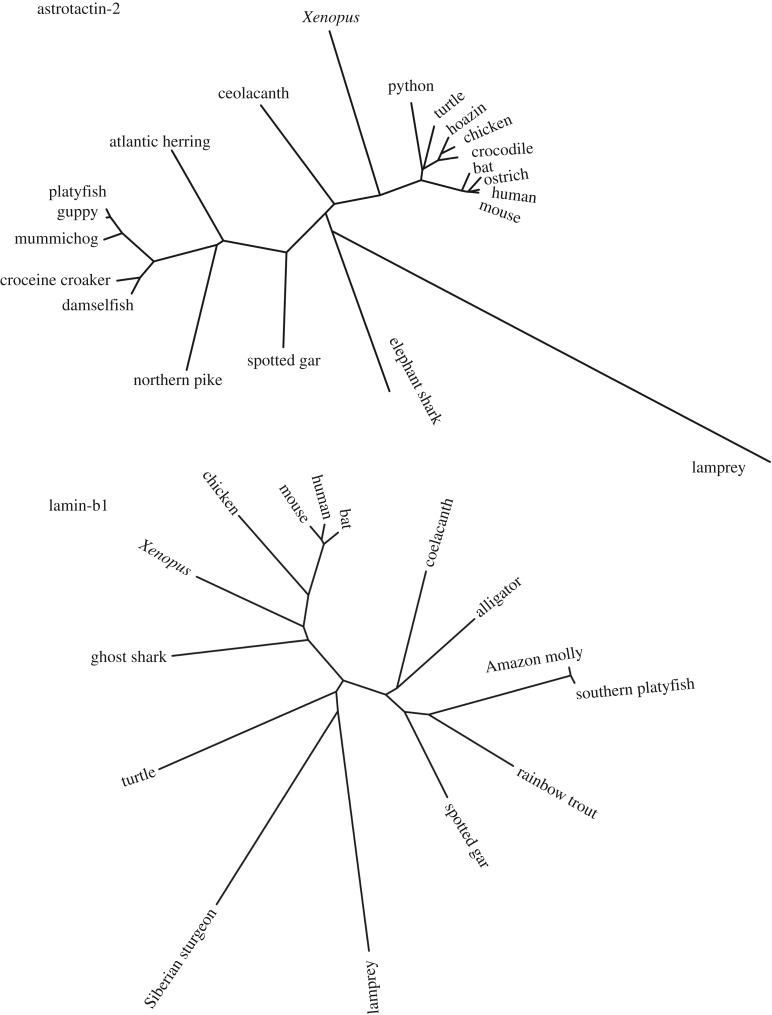
Phylogenetic tree of ASTNs and lamins across different species. The ASTN2 phylogeny is as reported previously [57]. For the lamin analysis, a lamprey lamin sequence (UniProtKB entry S4REH4) was used to probe the UniProtKB and top hits for representative species were taken and aligned in Clustal Omega leading to determination of a phylogenetic tree as shown. The phylogenetic tree was generated using FITCH and DRAWTREE as part of the PHYLIP package.

Structural phylogenetic analyses have played a useful role in marking out the ancient lineage and interrelationship of different PLPs [1]. For the purposes of discussing developmentally active PLPs, however, we here show sequence-based phylogenies for ASTN2 and, for comparison, lamins (figure 5). As previously described [57], ASTN2 in humans and in lampreys share 53% sequence identity, which is at least noteworthy given that they last shared a common ancestor 485 Myr ago. To place this in perspective, this was a point in evolution prior to the emergence of land animals or plants, and naturally means that all dinosaurs (to take a totemic example of an extinct animal set of species) possessed ASTNs. Perhaps the appearance of ASTNs was an adaptation of the Cambrian explosion—diversified, it seems likely, from their common ancestor with perforin-2. As also previously noted [57], the sequence diversity found among marine species is much greater than that found among land-dwelling vertebrates, which in our view has two likely origins: the more ancient lineage of the marine animals but, probably more significantly, the selective pressures of operating in a terrestrial environment having a more constraining effect on sequence variation.

For this review, we have compared the sequence-based phylogeny of ASTN2 with those for the lamins (figure 5). A lamin-like protein is thought to have been the common ancestor of intermediate filament proteins and they therefore represent a protein type which is fundamental to metazoan cell biology; while vertebrates commonly have two to four lamins, non-vertebrate metazoans often have only one [69,70]. The phylogeny of the vertebrate lamins compares interestingly with that of ASTN2. Lamins are more evenly divergent from the last common ancestor of all vertebrates than ASTN2 is and do not display the same sequential clustering within terrestrial species as opposed to marine animals. This suggests that the selective pressures acting on lamins that work within cells to structure basic functions are distinct from the selective pressures working on the ASTNs, with their role in neurodevelopment. It supports the idea previously advanced [57] that the processes of nervous system development on land might much more tightly constrain the variability tolerated in ASTNs than for similar processes occurring in water. Conversely, the lamins do not seem to experience a stronger selective pressure against variation in terrestrial as opposed to marine environments. The sequence comparison with the lamins does also, in a simple way, underscore the extraordinary conservation shown by the ASTNs: human and lamprey lamins B are 49% identical.

## 5. Conclusion and perspective

The developmental PLPs are an evolutionarily fascinating subgroup of MACPF family members. They show a unique combination of domains such as the modified Fn(III) domain resolved in ASTN2 and the annexin-like domain which clearly is homologous to human annexin V but shares no detectable sequence similarity. On the other hand, the sequence identity of the ASTNs is exceptional—to find 53% identity conserved over essentially the whole history of vertebrate life since the Cambrian explosion is striking and suggests the fundamental importance of the roles played by such proteins. Yet, we still do not know how the ASTNs and other related proteins perform their critical roles in development: in the next few years, we must hope that this will change and a proper mechanical and structural understanding of ASTN and BRINP biology will emerge. Just as the exceptional structural conservation of the MACPF domain in the first place suggests a capacity to deliver specific activities of selective advantage which have ensured its conservation since the last common ancestor of eubacteria and humans, so the exceptional sequence conservation of the ASTNs, for example, must indicate the importance of their role. We see that too in the implication of genetic variants associated with ASTN in a host of diseases from schizophrenia to Alzheimer's and can hope that the details of their highly conserved role in biology may help in the acquisition of an improved understanding of the underlying causes of such pathologies.
